# GCN5L1 impairs diastolic function in mice exposed to a high fat diet by restricting cardiac pyruvate oxidation

**DOI:** 10.14814/phy2.15415

**Published:** 2022-08-03

**Authors:** Dharendra Thapa, Paramesha Bugga, Bellina A. S. Mushala, Janet R. Manning, Michael W. Stoner, Brenda McMahon, Xuemei Zeng, Pamela S. Cantrell, Nathan Yates, Bingxian Xie, Lia R. Edmunds, Michael J. Jurczak, Iain Scott

**Affiliations:** ^1^ Vascular Medicine Institute Pittsburgh Pennsylvania USA; ^2^ Center for Metabolism and Mitochondrial Medicine, Department of Medicine University of Pittsburgh Pittsburgh Pennsylvania USA; ^3^ Biomedical Mass Spectrometry Center, Schools of the Health Sciences University of Pittsburgh Pittsburgh Pennsylvania USA; ^4^ Department of Cell Biology University of Pittsburgh Pittsburgh Pennsylvania USA; ^5^ Department of Chemistry University of Pittsburgh Pittsburgh Pennsylvania USA; ^6^ Division of Exercise Physiology West Virginia University School of Medicine Morgantown West Virginia USA

**Keywords:** acetylation, diastolic dysfunction, heart failure, mitochondria, pyruvate dehydrogenase

## Abstract

Left ventricular diastolic dysfunction is a structural and functional condition that precedes the development of heart failure with preserved ejection fraction (HFpEF). The etiology of diastolic dysfunction includes alterations in fuel substrate metabolism that negatively impact cardiac bioenergetics, and may precipitate the eventual transition to heart failure. To date, the molecular mechanisms that regulate early changes in fuel metabolism leading to diastolic dysfunction remain unclear. In this report, we use a diet‐induced obesity model in aged mice to show that inhibitory lysine acetylation of the pyruvate dehydrogenase (PDH) complex promotes energetic deficits that may contribute to the development of diastolic dysfunction in mouse hearts. Cardiomyocyte‐specific deletion of the mitochondrial lysine acetylation regulatory protein GCN5L1 prevented hyperacetylation of the PDH complex subunit PDHA1, allowing aged obese mice to continue using pyruvate as a bioenergetic substrate in the heart. Our findings suggest that changes in mitochondrial protein lysine acetylation represent a key metabolic component of diastolic dysfunction that precedes the development of heart failure.

## Graphical abstract

Left ventricular diastolic dysfunction is a structural and functional condition that precedes the development of heart failure with preserved ejection fraction (HFpEF). The etiology of diastolic dysfunction includes alterations in fuel substrate metabolism that negatively impact cardiac bioenergetics, and may precipitate the eventual transition to heart failure. In this report, we use a diet‐induced obesity model and acetylproteomics in aged mice to show that inhibitory lysine acetylation of the pyruvate dehydrogenase (PDH) complex promotes energetic deficits and diastolic dysfunction in mouse hearts.

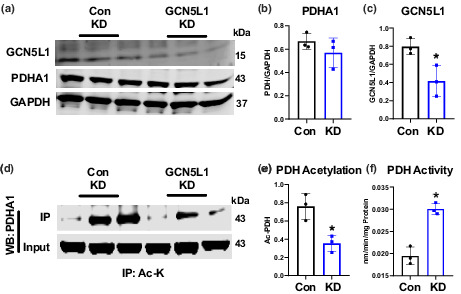

## 1 INTRODUCTION

Left ventricular diastolic dysfunction is an independent pre‐clinical predictor of all‐cause mortality (Redfield et al., 2003) and is a prerequisite for the diagnosis of heart failure with preserved ejection fraction (HFpEF) (Shah et al., 2017). While the etiology of the diastolic dysfunction is multifactorial, deleterious changes in cardiac energy metabolism underpin the development of both diastolic dysfunction and HFpEF (Lopaschuk et al., 2021; Ritchie & Abel, 2020). Studies on two recently developed murine models of HFpEF have reported maladaptive changes to the relative oxidation rates of fatty acids, ketones, and glucose in failing hearts (Deng et al., 2021; Tong, Schiattarella, et al., 2021), suggesting that this metabolic phenotype is central to the development of cardiac dysfunction. Interestingly, the HFpEF phenotype in these two independent mouse models is also characterized by an increase in mitochondrial protein acetylation, a reversible posttranslational modification that is often linked to altered metabolic enzyme activity (Deng et al., 2021; Tong, Schiattarella, et al., 2021). Restoring normal mitochondrial protein acetylation through supplementation with either nicotinamide riboside (to stimulate SIRT3‐mediated protein deacetylation) (Tong, Schiattarella, et al., 2021), or ketone bodies (which inhibit fatty acid uptake and utilization) (Deng et al., 2021), resulted in normalized cardiac bioenergetics and amelioration of the HFpEF phenotype (Deng et al., 2021; Tong, Schiattarella, et al., 2021).

These key studies suggest that tight regulation of mitochondrial protein acetylation is required to prevent the development of HFpEF, and therefore approaches that reduce maladaptive protein hyperacetylation are potentially cardioprotective. We have previously shown that diet‐induced increases in the abundance of a mitochondrial lysine acetylation regulatory protein, GCN5L1, correlated with increased fatty acid oxidation and reduced glucose utilization in the heart (Thapa et al., 2017, 2019). Here, using a mouse model of constitutively reduced mitochondrial protein acetylation, we examined the metabolic mechanisms underlying diastolic dysfunction in mice prior to the development of heart failure. Our results suggest that long‐term exposure to excess fat in aged mice promotes diastolic dysfunction in the heart, via inhibitory acetylation of enzymes involved in cardiac pyruvate oxidation.

## 2 METHODS

### 2.1 Transgenic mice

C57BL/6NJ wildtype (GCN5L1^WT/WT^, Cre^+/−^; “WT”) and cardiomyocyte‐specific inducible GCN5L1 knockout (GCN5L1^FL/FL^, Cre^+/−^; “KO”) mice used in the studies were generated as previously reported (Thapa, Manning, Stoner, et al., 2020). GCN5L1 deletion in cardiomyocytes was induced via single tamoxifen injection (40 mg/kg IP) and validated as previously described (Thapa, Manning, Stoner, et al., 2020).

### 2.2 Animal care and experimental diets

Male WT and GCN5L1 KO animals aged 5–7 months were fed either a standard low fat diet (LFD; Research Diets D12450B), or a high fat diet (HFD; Research Diets D12492), for 30 weeks. Mice were euthanized by CO_2_ asphyxiation and rapid cervical dislocation. All animal procedures were approved by the University of Pittsburgh Institutional Animal Care and Use Committee.

### 2.3 Ultrasonography

Animals were anesthetized using isofluorane (1.5%–2.0% v/v by inhalation) and monitored for cardiac functional parameters using a VisualSonics Vevo 3100 in M‐mode echocardiography, Tissue Doppler, and Pulsed‐Wave Doppler modes. Markers of systolic and diastolic function were calculated using standard echocardiography equations.

### 2.4 Respirometry

Respirometry was performed using an Oroboros O2K High‐Resolution Respirometer using mitochondrially enriched cardiac samples. Respiration was measured in MiR05 buffer at 37°C under constant mixing in a sealed, 2 ml chamber. The respirometry protocol consisted of sequential additions of substrates and inhibitors as follows: pyruvate (5 mM) or BSA‐conjugated palmitate (0.25 mM), malate (2 mM), ADP (2 mM); oligomycin (2.5 μM); carbonyl cyanide 4‐(trifluoromethoxy)phenylhydrazone (FCCP; titrations of 0.5 μM until maximal respiration reached); antimycin A (2.5 μM). Protein concentration of the mitochondrially enriched fraction was determined by BCA protein assay, and oxygen consumption or flux expressed per mg protein normalized to citrate synthase activity.

### 2.5 Biochemical assays

PDH activity was assessed using a commercial kit (MAK‐183; Sigma‐Aldrich) according to the manufacturer's instructions.

### 2.6 Cell culture and transfection

Stable control shRNA and GCN5L1 shRNA AC16 cells were described previously (Manning et al., 2019). WT PDHA1‐Myc, 5KR PDHA1‐Myc, and 5KQ PDHA1‐Myc were produced using custom gene synthesis (Genscript), and cloned into the pCMV‐3Tag‐4a plasmid vector. Cells were transfected for 24 h using X‐tremeGENE reagent.

### 2.7 Protein isolation, western blotting, and immunoprecipitation

For western blotting, tissues were minced and lysed in CHAPS buffer (1% CHAPS, 150 mM NaCl, 10 mM HEPES, pH 7.4) on ice for ~2 h. Homogenates were spun at 10,000 *g*, and supernatants were collected. Protein lysates were prepared in LDS sample buffer, separated using Bolt SDS/PAGE 4%–12% or 12% Bis‐Tris gels, and transferred to nitrocellulose membranes (all Life Technologies). Protein expression was analyzed using the following primary antibodies; rabbit PDHA1 Ser‐293 antibody (Cell Signaling; #31866), rabbit PDHA1 (Cell Signaling; #3205), and rabbit GAPDH (Cell Signaling; #2118). GCN5L1 antibody was made by Covance and previously validated (Thapa, Manning, Stoner, et al., 2020). Fluorescent anti‐mouse or anti‐rabbit secondary antibodies (red, 700 nm; green, 800 nm) from Li‐Cor were used to detect expression levels. Protein densitometry was measured using Image J software (National Institutes of Health). For immunoprecipitation experiments, tissues were minced and lysed in CHAPS buffer (1% CHAPS, 150 mM NaCl, 10 mM HEPES, pH 7.4) on ice for ~2 h. Homogenates were spun at 10,000 *g*, and supernatants were collected. Protein lysates were incubated overnight at 4°C with rabbit acetyl‐lysine (Ac‐K, #9441) from Cell Signaling Technology. Immunocaptured proteins were isolated using Protein‐G agarose beads (Cell Signaling Technology, #9007), washed multiple times with CHAPS buffer, and then eluted in LDS sample buffer (Life Technologies) at 95°C. Samples were separated on 12% Bis‐Tris Bolt gels and probed with appropriate antibodies. Protein densitometry was measured using Image J software (National Institutes of Health).

### 2.8 Statistics

Graphpad Prism software was used to perform statistical analyses. Means ± SEM were calculated for all data sets. Data were analyzed using two‐way ANOVA with Tukey's post‐hoc multiple comparison testing to determine differences between genotypes and feeding groups. Data were analyzed with two‐tailed Student's *t*‐tests to determine differences between single variable groups. *p <* 0.05 was considered statistically significant.

## 3 RESULTS

### 3.1 Cardiomyocyte‐specific GCN5L1 cKO mice are resistant to diastolic dysfunction induced by a high fat diet

While the two HFpEF mouse studies referenced above used diet‐based two‐hit (Tong, Schiattarella, et al., 2021) or three‐hit (Deng et al., 2021) models to produce the HFpEF phenotype, long‐term exposure to a high fat diet (HFD) alone can promote diastolic dysfunction (Tong, Saito, et al., 2021). We therefore used a long‐term HFD model in aged mice to specifically examine diastolic dysfunction in a pre‐HFpEF state. Wildtype (WT) and cardiomyocyte‐specific GCN5L1 knockout (KO) mice (which display global reductions in mitochondrial protein acetylation) (Thapa, Manning, Stoner, et al., 2020) were placed on low fat (LFD; 10% fat) or high fat (HFD; 60% fat) diets for 30 weeks, and heart structure and function were measured by ultrasonography (Table 1). Consistent with previous studies, no differences in body weight were observed in WT or KO mice on either a LFD or HFD (data not shown) (Thapa, Manning, Stoner, et al., 2020). No significant changes in left ventricle systolic function (LVEF) were observed between WT and KO mice on either a LFD or HFD (Table 1). While the E/e' ratio (a marker of diastolic dysfunction) in KO mice was mildly (but non‐significantly) elevated relative to WT mice under LFD conditions (20 ± 3 vs. 24 ± 5, *p* = 0.35), KO mice did not display any further increase in diastolic dysfunction when placed on a HFD for 30 weeks (Table 1). In contrast, WT mice developed diastolic dysfunction (as shown by an elevated E/e' ratio) after 30 weeks of HFD diet relative to WT mice on a LFD (Table 1). Combined, these data suggest that GCN5L1 expression in cardiomyocytes promotes the development of diastolic dysfunction, but not systolic dysfunction, when aged mice are exposed to a HFD for an extended period.

**TABLE 1:** GCN5L1 promotes diastolic dysfunction in aged mice after exposure to a long‐term high fat diet

Parameter	WT LFD	KO LFD	WT HFD	KO HFD
Heart rate (BPM)	463 ± 51	476 ± 40	494 ± 50	475 ± 22
Volume (s; μl)	28 ± 11	20 ± 8	31 ± 6	38 ± 20
Volume (d; μl)	67 ± 13	62 ± 28	79 ± 15	93 ± 39
LV mass (mg)	140 ± 27	159 ± 44	179 ± 27*	238 ± 80
Cardiac output (ml/min)	19 ± 5	20 ± 11	23 ± 5	26 ± 10
LV AW (s; mm)	1.2 ± 0.2	1.5 ± 0.1	1.3 ± 0.1	1.5 ± 0.1
LV AW (d; mm)	0.9 ± 0.1	1.1 ± 0.1	1.1 ± 0.1*	1.2 ± 0.1
LV PW (s; mm)	1.2 ± 0.3	1.4 ± 0.2	1.4 ± 0.2	1.5 ± 0.2
LV PW (d; mm)	0.9 ± 0.2	1.0 ± 0.1	1.0 ± 0.1	1.1 ± 0.1
Stroke volume (μl)	40 ± 8	42 ± 21	48 ± 11	54 ± 20
LV EF (%)	60 ± 12	67 ± 7	61 ± 5	60 ± 6
LV FS (%)	31 ± 8	37 ± 6	32 ± 4	32 ± 4
E (mm/s)	521 ± 65	636 ± 41	645 ± 87*	672 ± 101
e' (mm/s)	27 ± 4	27 ± 5	24 ± 4	28 ± 6
E/e' (ratio)	20 ± 3	24 ± 5	28 ± 5*	26 ± 5

*Note*: Ultrasonography measurements of left ventricle structure and function in wildtype (WT) and cardiomyocyte‐specific GCN5L1 knockout (KO) mice after 30 weeks of low fat diet (LFD) or high fat diet (HFD). Values presented are mean ± SEM.

* *p* < 0.05 vs WT LFD.

### 3.2 GCN5L1 cKO mice have enhanced cardiac pyruvate oxidation capacity under high fat diet conditions

**FIGURE 1:**
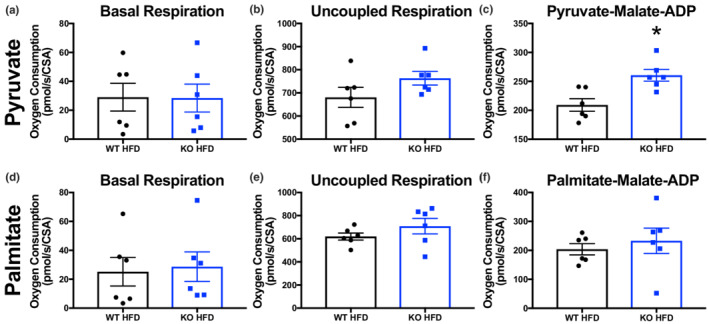
GCN5L1 inhibits cardiac pyruvate oxidation in obese mice. (a–f) oxygen consumption in cardiac tissues from HFD‐fed WT and KO mice was measured in response to either pyruvate‐malate‐ADP (a–c) or palmitate‐malate‐ADP (d–f) using Oroboros respirometry. *N* = 6; *t*‐test; *p < 0.05.

Alterations in fuel substrate metabolism are central to the development of diastolic dysfunction (Deng et al., 2021; Tong, Schiattarella, et al., 2021). To understand which fuel metabolism pathways were disrupted in mice displaying diastolic dysfunction, we performed Oroboros respirometry in cardiac tissue lysates from HFD‐fed WT and KO mice. Previous reports have shown that fatty acid oxidation is significantly modified in hyperacetylated mitochondria from HFpEF‐model mice (Deng et al., 2021; Tong, Schiattarella, et al., 2021). However, we found here that ADP‐stimulated state 3 respiration was not significantly different between HFD WT and KO samples provided with palmitate as the fuel substrate (Figure 1). Instead, we observed a significant increase in ADP‐stimulated state 3 respiration in HFD KO tissues given pyruvate as a fuel source, relative to their WT counterparts on the same diet (Figure 1). Combined, these data indicate that a loss of pyruvate oxidation, rather than aberrant fatty acid oxidation, underpins the energetic deficits that may contribute to the development of diastolic dysfunction in this model.

### 3.3 Exposure to a high fat diet inhibits pyruvate oxidation through hyperacetylation of PDHA1

**FIGURE 2:**
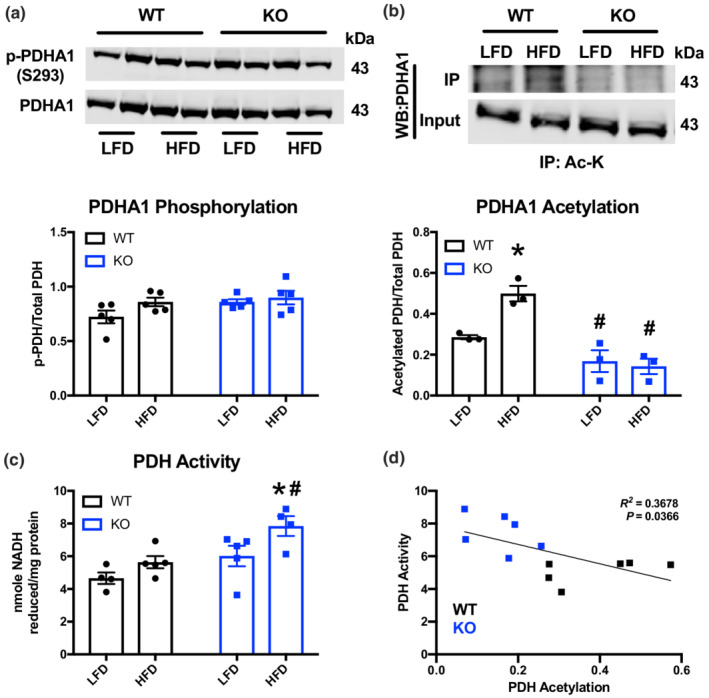
GCN5L1 promotes PDHA1 acetylation and inhibits pyruvate dehydrogenase activity. (a, b) PDHA1 phosphorylation (S293) and lysine acetylation in WT and KO mice following LFD or HFD. *N* = 3–5; two‐way ANOVA; *p < 0.05 relative to WT LFD, ^#^ *p* < 0.05 to WT HFD (c) PDH activity in WT and KO mice following LFD or HFD. *N* = 3–5; two‐way ANOVA; *p < 0.05 relative to WT LFD, ^#^ *p* < 0.05 to WT HFD. (d) Linear regression of PDH activity and acetylation levels in WT and KO mice following LFD or HFD.

The pyruvate dehydrogenase (PDH) complex regulates mitochondrial pyruvate utilization, and previous work has demonstrated that interventions that increase PDH activity can reverse diastolic dysfunction in diabetic rodents (Gopal et al., 2021; Le Page et al., 2015). As such, we examined whether GCN5L1 had a regulatory effect on PDH activity following exposure to a long‐term HFD. Elevated PDH activity is commonly associated with a decrease in inhibitory phosphorylation at Ser‐293(Tong, Schiattarella, et al., 2021); however, we detected no difference in PDH phosphorylation between WT and KO mice on either diet (Figure 2a). Instead, we found a significant increase in the acetylation status of PDHA1 (a component of the PDH E1 enzyme) in HFD‐fed WT mice relative to LFD controls, and a significant decrease in PDHA1 acetylation in both KO diet groups (Figure 2b). Consistent with this, PDH activity was significantly increased in KO mice on a long‐term HFD relative to WT mice (Figure 2c), and PDHA1 acetylation status across all animal groups was negatively correlated with PDH activity by linear regression (*R* ^2^ = 0.3678; *p* = 0.037; Figure 2d). Combined, these data suggest that exposure to a long‐term HFD in aged mice inhibits PDH through increased lysine acetylation, rather than through the more common phosphorylation inhibitory pathway.

### 3.4 GCN5L1 expression correlates with reduced PDH activity in cultured cardiac cells

**FIGURE 3:**
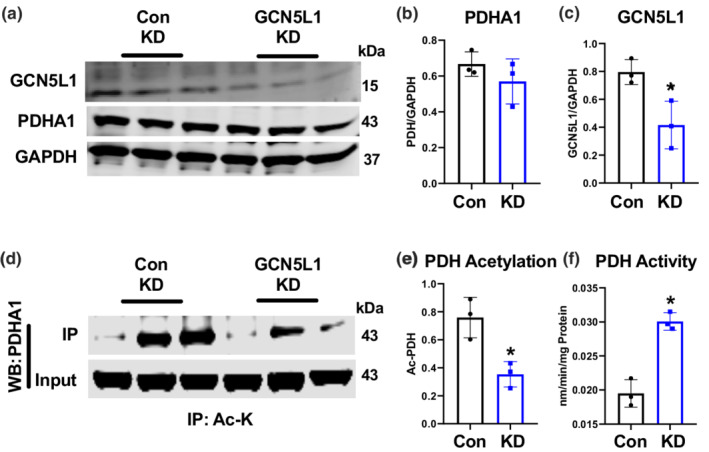
GCN5L1 expression correlates with reduced PDH activity in cardiac cells. (a–f) Total PDH acetylation and enzymatic activity was measured in stable control shRNA or GCN5L1 shRNA knockdown (KD) human cardiac AC16 cells. *N* = 3; *t*‐test; *p < 0.05 relative to WT.

To verify these findings independently, we examined PDH activity and acetylation status in the human‐derived AC16 cardiomyocyte cell model following shRNA‐mediated GCN5L1 depletion. Knockdown of GCN5L1 in AC16 cells resulted in a ~50% decrease in PDHA1 acetylation, and a ~40% increase in PDH activity, without observed changes in total PDH abundance (Figure 3a–f). Combined with our in vivo findings, these data suggest that GCN5L1‐mediated hyperacetylation of PDH is a key driver of reduced pyruvate oxidation in cardiac cells.

### 3.5 Preventing PDHA1 acetylation promotes pyruvate dehydrogenase activity in cardiac cells

**FIGURE 4:**
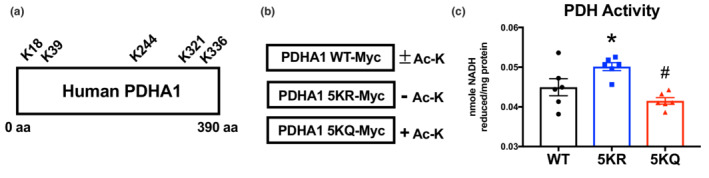
Acetylation of PDHA1 directly reduces its enzymatic activity. (a) Schematic of human PDHA1 showing published lysine acetylation sites. (b, c) GCN5L1 KD AC16 cells were transfected with Myc‐tagged wildtype PDHA1 (WT‐Myc), a non‐acetylated PDHA1 mimic in which lysines K18/K39/K244/K321/K336 were replaced with arginine (5KR‐Myc), and an acetylated PDHA1 mimic where lysines K18/K39/K244/K321/K336 were replaced with glutamine (5KQ‐Myc). *N* = 6; one‐way ANOVA; *p < 0.05 relative to WT, ^#^ *p* < 0.05 to 5KR.

Finally, we sought to determine the specific effect of lysine hyperacetylation on PDH enzymatic activity. Published studies in the PhosphoSitePlus proteomics database (www.phosphosite.org) have identified five acetylation sites on human PDHA1 at K18, K39, K244, K321, and K336 (Figure 4a). We generated novel PDHA1 expression constructs (Figure 4b; Figure S1) where each of these five lysines were replaced with either arginine (5KR; to mimic a non‐acetylated state) or glutamine (5KQ; to mimic a hyperacetylated state), and expressed them in GCN5L1 knockdown AC16 cells (which represent a basal, non‐acetylated state for endogenous PDHA1). PDH activity was significantly increased in non‐acetylated PDHA1 5KR expressing cells relative to those overexpressing wildtype PDHA1 (Figure 4c). Conversely, PDH activity was significantly decreased in hyperacetylated PDHA1 5KQ expressing cells relative to PDHA1 acetylation‐deficient 5KR expressing cells (Figure 4c). Together, these in vitro data indicate that hyperacetylation of PDHA1 has a specific negative effect on PDH enzymatic activity in cardiac cells, which matches the effect of global PDHA1 hyperacetylation in obese mice with diastolic dysfunction.

## 4 DISCUSSION

Previous work from our group and others has demonstrated that increased mitochondrial protein acetylation in the heart is linked with increased rates of fatty acid oxidation ex vivo and in vitro (Alrob et al., 2014; Fukushima et al., 2016; Thapa et al., 2017). However, the functional consequence of this change is unclear, as increased fatty acid oxidation activities in hyperacetylated hearts did not directly lead to increased contractility under ex vivo conditions (Thapa, Manning, Mushala, et al., 2020). Results from the current study are instead in agreement with previous work showing that PDH acetylation is linked to a decrease in its activity in the heart in vivo and ex vivo (Mori et al., 2013; Thapa et al., 2019), and that GCN5L1 overexpression leads to elevated PDH acetylation in vivo and in vitro (Thapa et al., 2019). This is the first loss‐of‐function study showing that GCN5L1 regulates the acetylation status of PDHA1 in vivo, and demonstrates that increased abundance of this modification is linked to both decreased pyruvate utilization, and impaired diastolic function, in the hearts of aged obese mice. Our work suggests that manipulation of PDHA1 acetylation levels in vivo may represent a novel target for therapeutic intervention in the treatment of diastolic dysfunction.

The inhibitory effect of PDH acetylation in diastolic dysfunction described here is consistent with its role in mouse models of heart failure with reduced ejection fraction (HFrEF), where miR‐195‐mediated downregulation of the deacetylase SIRT3 leads to increased acetylation of PDH subunits, decreased PDH enzymatic activity, and systolic dysfunction (Zhang et al., 2018). However, in this study we detected no differences in SIRT3 abundance in response to diet or genotype (Figure S2), suggesting that GCN5L1 instead is the main driver of changes in PDH acetylation in our diastolic dysfunction model. This finding is consistent with previous work showing that exposure to a high fat diet increases GCN5L1 expression and PDH acetylation in the heart (Thapa et al., 2017; Thapa, Manning, Stoner, et al., 2020), and through loss‐of‐function studies confirms a direct effect of GCN5L1 activity on PDHA1 acetylation status.

Interestingly, the deletrious effect of global mitochondrial protein hyperacetylation in the development of HFpEF (Deng et al., 2021; Tong, Schiattarella, et al., 2021) sits at odds with its demonstrated lack of effect in mouse surgical models of HFrEF (Davidson et al., 2020; Scott & Sack, 2020). In that study, mice with a combined genetic deletion of the deacetylase, SIRT3, and the carnitine acetyltransferase, CrAT, did not show increased susceptibility to pressure overload‐induced heart failure despite massively hyperacetylated mitochondria (Davidson et al., 2020). Further work will be required to determine whether the differences found reflect the etiology of the different models used (i.e. nutrition vs. surgical pressure overload); the site‐specific nature of the acetylation sites modified on each protein (i.e. whether lysine residues on the same protein are differentially regulated by the two models); or whether the different mechanisms by which GCN5L1 and SIRT3 control mitochondrial protein acetylation status (acetyl‐CoA dependent acetylation vs. NAD^+^ dependent deacetylation), lead to different functional outcomes.

Inhibition of PDH activity has previously been shown to drive the development of diastolic dysfunction, and interventions that relieve this inhibition have focused on downregulating the activity of the PDH kinase, PDK4. These interventions include the use of the PDK4 inhibitor dichloroacetate (Le Page et al., 2015), and chemical inhibition of FoxO1 transcriptional activity (which drives increased PDK4 expression in type 2 diabetes) (Gopal et al., 2021), both of which improved diastolic function in diabetic rodents. In this study, we found that changes in PDH acetylation status correlated with PDH enzymatic activity without detectable changes in PDH phosphorylation (Figure 2). While the current study used aging and diet to generate the mild diastolic dysfunction observed, the two studies mentioned above generated a diabetic phenotype by combining a high fat diet with streptozotocin treatment to disrupt β‐cell function (Gopal et al., 2021; Le Page et al., 2015). Future studies will be required to determine whether the aging component, or the chemical induction of diabetes, drives the difference in PDH regulation seen in these models.

**FIGURE 5:**
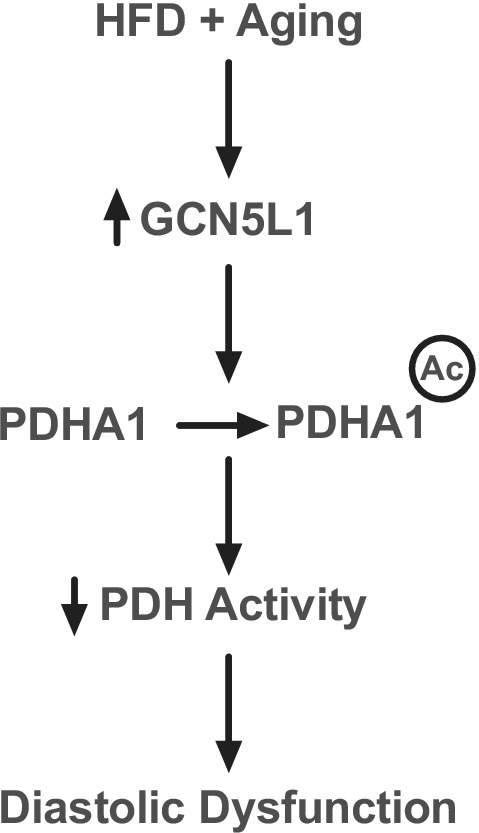
Schematic of proposed PDH regulation by GCN5L1. A combination of nutrient excess and aging leads to increased GCN5L1 expression (Thapa et al., 2017; Thapa, Manning, Stoner, et al., 2020), which results in significantly increased PDHA1 lysine acetylation. Increased PDHA1 acetylation results in decreased PDH enzymatic activity, which contributes to the diastolic dysfunction observed in obese, aged mice.

In conclusion, we show that inhibitory acetylation of PDHA1 drives reduced pyruvate utilization in a diet‐based model of mild cardiac diastolic dysfunction (Figure 5). Mice lacking cardiomyocyte expression of the mitochondrial acetylation regulator GCN5L1 are protected from diastolic dysfunction in response to long‐term nutritional excess, potentially due to an absence of inhibitory PDHA1 acetylation. These findings corroborate previous work showing the decreased PDH activity is a causative factor in the development of diastolic dysfunction (Gopal et al., 2021; Le Page et al., 2015), and describe a new potential mechanism underlying this disease process in aged mice.

## AUTHOR CONTRIBUTIONS

Performed experiments: DT, PB, BASM, JRM, MWS, BM, XZ, PC, BX, LRE. Designed experiments: DT, PB, NY, MJJ, IS. Analyzed data: DT, PB, BM, XZ, PC, NY, BX, LRE, MJJ, IS. Produced figures: DT, PB, IS. Wrote/edited manuscript: DT, PB, NY, MJJ, IS.

## FUNDING INFORMATION

This work was supported by: NIH/NHLBI K99/R00 (HL146905) to DT; NIGMS T32 (GM133332) to B.A.S.M; and NIH/NHLBI R01 (HL132917 & HL147861) research grants to IS. The University of Pittsburgh Center for Metabolism and Mitochondrial Medicine is supported by a Pittsburgh Foundation (MR2020 109502) grant to MJJ. The University of Pittsburgh Rodent Ultrasonography Core received support from the NIH/OD S10 Instrumentation Program (OD023684).

## DISCLOSURES

None.

## ETHICS STATEMENT

The studies reported in this manuscript were approved by the relevant regulatory bodies at the University of Pittsburgh.

## Supporting information


Figure S1
Figure S2
