# Supplementary material for: GCN5L1 impairs diastolic function in mice exposed to a high fat diet by restricting cardiac pyruvate oxidation
Source: Physiol Rep. 2022 Aug 3;10(15):e15415. doi: 10.14814/phy2.15415 (PMC9350469; doi:10.14814/phy2.15415)
Supplement: Supplementary file 1 — Figure S1 Figure S2 [file PHY2-10-e15415-s001.docx]

**SUPPLEMENTAL INFORMATION**

**Supplemental Methods**

**Quantitative Proteomics**

Flash-frozen left ventricle tissues were homogenized in sodium deoxycholate (SDC) lysis buffer using a bead mill (3 x 60 s at 6.5 s/m), and sonicated for 15 minutes in a water bath to shear DNA. Extracted proteins (1.5 mg) were reduced (DTT) and alkylated (CAA), the SDC buffer removed by acid precipitation, and desalted with Sep-Pek C18. After in-solution trypsin digest, peptides were desalted using PepClean C18 (Pierce) and subject to LC/MS/MS. Data were analyzed using the MaxQuant software suite to measure SIRT3 abundance.

**Supplemental Figures**

**Supplemental Figure 1: Representative blot of PDHA1 construct overexpression.** WT and mutant PDHA1 constructs were expressed in AC16 cells, and detected by immunoblotting for PDHA1.

**Supplemental Figure 2: Proteomic quantification of SIRT3 abundance.** No change was observed in the abundance of SIRT3 in in either diet (LFD vs. HFD) or genetic (WT vs. GCN5L1 KO) models.
